# Effects of Simvastatin Beyond Dyslipidemia: Exploring Its Antinociceptive Action in an Animal Model of Complex Regional Pain Syndrome-Type I

**DOI:** 10.3389/fphar.2017.00584

**Published:** 2017-09-04

**Authors:** Graziela Vieira, Juliana Cavalli, Elaine C. D. Gonçalves, Tainara R. Gonçalves, Larissa R. Laurindo, Maíra Cola, Rafael C. Dutra

**Affiliations:** ^1^Laboratory of Autoimmunity and Immunopharmacology (LAIF), Department of Health Sciences, Center of Araranguá, Federal University of Santa Catarina Araranguá, Brazil; ^2^Post-Graduate Program of Cellular Biology and Developmental, Center of Biological Sciences, Federal University of Santa Catarina Florianópolis, Brazil; ^3^Post-Graduate Program of Neuroscience, Center of Biological Sciences, Federal University of Santa Catarina Florianópolis, Brazil

**Keywords:** simvastatin, inflammatory pain, neuropathic pain, ischemia-reperfusion, TRPM8 channel

## Abstract

Simvastatin is a lipid-lowering agent that blocks the production of cholesterol through inhibition of 3-hydroxy-methyl-glutaryl coenzyme A (HMG-CoA) reductase. In addition, recent evidence has suggested its anti-inflammatory and antinociceptive actions during inflammatory and pain disorders. Herein, we investigated the effects of simvastatin in an animal model of complex regional pain syndrome-type I, and its underlying mechanisms. Chronic post-ischemia pain (CPIP) was induced by ischemia and reperfusion (IR) injury of the left hind paw. Our findings showed that simvastatin inhibited mechanical hyperalgesia induced by CPIP model in single and repeated treatment schedules, respectively; however simvastatin did not alter inflammatory signs during CPIP model. The mechanisms underlying those actions are related to modulation of transient receptor potential (TRP) channels, especially TRMP8. Moreover, simvastatin oral treatment was able to reduce the nociception induced by acidified saline [an acid-sensing ion channels (ASICs) activator] and bradykinin (BK) stimulus, but not by TRPA1, TRPV1 or prostaglandin-E2 (PGE2). Relevantly, the antinociceptive effects of simvastatin did not seem to be associated with modulation of the descending pain circuits, especially noradrenergic, serotoninergic and dopaminergic systems. These results indicate that simvastatin consistently inhibits mechanical hyperalgesia during neuropathic and inflammatory disorders, possibly by modulating the ascending pain signaling (TRPM8/ASIC/BK pathways expressed in the primary sensory neuron). Thus, simvastatin open-up new standpoint in the development of innovative analgesic drugs for treatment of persistent pain, including CRPS-I.

## Introduction

Statins or HMG-CoA reductase inhibitors act on the rate-limiting step in cholesterol biosynthesis representing an established therapy for prevention of dyslipidemia and coronary artery diseases (Zhou and Liao, 2009). Moreover, clinical findings suggest that the broad benefits observed with statins may not be mediated exclusively by their lipid-lowering properties, but possibly through cholesterol-independent or pleiotropic effects. Several evidences have shown that statins present a range of pleiotropic effects, such as anti-inflammatory (Otuki et al., 2006; Montecucco and Mach, 2009; Adami et al., 2012; Tabas and Glass, 2013), immunomodulatory (Greenwood et al., 2006; Krysiak et al., 2016), anti-periodontitis (Machado et al., 2014), neuroprotective (van der Most et al., 2009; Chataway et al., 2014), antioxidant (Margaritis et al., 2014), anticancer (Nielsen et al., 2012), antibiotic (Hennessy et al., 2013), and antidepressant (Kim et al., 2015; Köhler-Forsberg et al., 2017), as well as antinociceptive effects. From the 2010s, statin – including simvastatin and atorvastatin – antinociceptive effects have been demonstrated in different pre-clinical models of pain (Garcia et al., 2011; Miranda et al., 2011; Shi et al., 2011) and it has been suggested that mechanism by which simvastatin induced its antinociception effect was attenuating the sensitization of spinal nociceptive transmission (Ohsawa et al., 2012), although its exact mechanism of action remains still unclear.

Primarily know as Sundeck’s dystrophy or causalgia, the complex regional pain syndrome (CRPS) is a disabling and distressing pain condition with spontaneous and stimulus-evoked pain, motor dysfunction, oedema, vasomotor and sudomotor abnormalities and trophic changes (Stanton-Hicks et al., 1995; Bruehl et al., 2002). Commonly, this condition resolves within the first year, with a smaller subset progressing to the neurogenic neuroinflammation form. This transition is often paralleled by a change from “warm complex regional pain syndrome,” with inflammatory characteristics dominant, to “cold complex regional pain syndrome” in which autonomic features dominate (Bruehl, 2015). In accordance to the International Association for the Study of Pain (IASP), CRPS is sub-divided in CRPS type I (CRPS-I) when there is no nerve injury and CRPS type II (CRPS-II) related to cases with nerve injury (Maihöfner et al., 2010). In order to study the physiopathology of the CRPS-I and its possible treatments, Coderre et al. (2004) developed the CPIP model, the most used tool employed to study the CRPS-I in laboratory, which produces a neuropathic-like pain syndrome in rodents following prolonged hindpaw I/R. In addition, rats display spontaneous pain behaviors (hindpaw shaking, licking and favoring) and spread of hyperalgesia/allodynia to the uninjured contralateral hindpaw (Coderre et al., 2004).

CRPS-I is recognized as being difficult to treat, despite of the treatment methods that are available, including physiotherapy, sympathetic blockade, corticosteroids, and non-steroidal anti-inflammatory drugs (Hord and Oaklander, 2003), which showed only moderate effects. Consequently, developing new pharmacological treatments, as well as investigating the mechanism of drugs already approved to other purposes has become fundamental to reach new therapies and innovative pharmacological strategies. Thus, according to the aforementioned background, the present study was delineated to investigate the simvastatin antinociceptive effect and its mechanism of action related to the ascending and descending pain control.

## Materials and Methods

### Animals

The experiments were carried out in male Swiss mice (20–30 g) obtained from the Federal University of Santa Catarina. The animals were kept under a 12 h light/dark cycle (lights on at 7:00 a.m.) and temperature (22 ± 2°C) with food and water *ad libitum* (maximum of 10 mice group-housed). Animals were acclimatized to the laboratory settings for at least 1 h before testing and were used only once throughout the experiments. Mice were randomly assigned before treatment or behavioral evaluation. All procedures used in the present study followed the “Principles of laboratory animal care” (NIH publication no. 85–23) and were approved by the Animal Ethics Committee of the Universidade Federal de Santa Catarina (CEUA-UFSC, protocol number PP00956). Moreover, the number of animals and the intensity of noxious stimuli used were the minimum necessary to demonstrate consistent effects. This study strictly followed the Arrive guidelines as previously reported by Kilkenny et al. (2010).

### Chronic Post-ischemia Pain Induction

Chronic post-ischemia pain was induced by IR injury of the left hindpaw, as described by Coderre et al. (2004). Concisely, animals were anesthetized over a 3-h period with a bolus (7%, 0.6 ml/kg, i.p.) of chloral hydrate (VETEC, São Paulo, Brazil) and 20% of the initial volume at the end of the first and second hour (Martins et al., 2013). After induction of anesthesia, a nitrile 70 durometer O-ring (O-rings West, Seattle, WA, United States) with a 7/32 internal diameter was placed around the mice’s left hind limb just proximal to the ankle joint. The termination of anesthesia was timed so that mice recovered fully within 30–60 min following reperfusion, which occurred immediately after removal of the O-ring. After 3 h the O-ring was cut, allowing reperfusion of the hind limb.

### Experimental Design

To investigate simvastatin effect on the neuropathic stage of CRPS-I, the mechanical hyperalgesia was evaluated at 7, 14, and 21 days after nerve injury. Simvastatin was administered in single treatment (5, 10, 50, and 100 mg/kg, sole administration during evaluated days) or repeated (50 mg/kg) treatment schedule, 1x/day by oral route (p.o.) during 21 days. Imipramine (20 mg/kg, i.p.) was used as positive control drug. In regard to simvastatin action on the inflammatory stage of CRPS-I, paw oedema, paw temperature, and mechanical hyperalgesia – day 1, 2, and day 3 – were evaluated in the naïve group, untreated I/R group, and in mice treated daily with simvastatin (50 mg/kg, 1x/day, p.o.).

To further investigate the mechanisms underlying the simvastatin actions, we assessed its effects on: (i) nociception and paw oedema response induced by intraplantar injection of menthol (1.2 nmol/paw; TRPM8 activator), cinnamaldehyde (10 nmol/paw; TRPA1 activator) and capsaicin (5.2 nmol/paw; TRPV1 activator); and (ii) licking and paw oedema response induced by intraplantar injection of acidified saline (pH 3/paw; an ASIC activator), BK (3 nmol/paw; BK pathway), and PGE2 (3 nmol/paw; prostaglandin pathway), according to previously described (Mantovani et al., 2006; Pigatto et al., 2016; Simões et al., 2016). In both conditions, the animals were pre-treated with simvastatin (50 mg/kg, p.o.) or vehicle (saline solution, 10 ml/kg, p.o., control group) 1 h before the algogenic injections.

Regarding underlying mechanisms of analgesia, we next evaluated some of the descending pain circuits, especially noradrenergic, serotoninergic and dopaminergic systems, involved in simvastatin antinociception effects. Thus, mice received an intraperitoneal (i.p.) pre-treatment with prazosin (0.15 mg/kg, a selective α1-adrenergic receptor antagonist), ketanserin (1 mg/kg, a selective 5-HT2A receptor antagonist) or haloperidol (1 mg/kg, a selective D2 receptor antagonist) 20 min prior to the oral administration of simvastatin (50 mg/kg) during menthol-induced paw nociception (DalBó et al., 2006; Mantovani et al., 2006).

### Mechanical Hyperalgesia

Mice were placed individually in clear plexiglas boxes (9 × 7 × 11 cm) on elevated wire mesh platforms allowing access to right hind paw ventral surface. Withdrawal response frequency was measured following 10 applications (1 s each) of von Frey filament (VFH; Stoelting, Chicago, IL, United States), as previously described (Quintão et al., 2005). VFH filament (0.6 g) was set to produce a mean withdrawal frequency of about 30%. The animals were acclimatized for at least 1 h before behavioral testing, and to determine the basal tactile thresholds, all the groups were evaluated before CPIP induction. Mechanical hyperalgesia was then accessed on 7, 14, and 21 days after nerve injury.

### Hyperaemia and Oedema

To access inflammatory signs (hyperaemia and oedema), which appear just after I/R injury, paw oedema and paw temperature were measured before and from 10 min to 8 h after the removal of the O-ring. On the 3 next following days (day 1, 2, and 3) after I/R injury, besides paw oedema and paw temperature, mechanical hyperalgesia were also measured. Hyperaemia was examined by measuring the temperature of the plantar surface of the hindpaw using a thermocouple probe connected to a transducer (BAT-12, Physitemp, Clifton, NJ, United States). Hindpaw oedema formation was described as Δpaw thickness = test paw thickness–basal paw thickness; paw thickness was measured using a digital micrometer (Digimess, São Paulo, Brazil). Both, temperature and oedema measurements, were based on an average of three replicate recordings taken at different time points between 30 min and 4 h after reperfusion.

### Materials

Simvastatin, menthol, capsaicin, cinnamaldehyde, PGE2, BK, haloperidol, prazosin hydrochloride and ketanserin (+)-tartrate salt were purchased from Sigma Chemical Co. (St. Louis, MO, United States). Acetic acid was obtained from Merck (Frankfurt, Darmstadt, Germany). Simvastatin was diluted in saline (0.9% NaCl solution) and administered orally by gavage (p.o.). The dilutions of the substances used in the experiments were: menthol (2% ethanol/3% Tween 80 in saline), capsaicin (10% ethanol/10% Tween 80 in saline) and cinnamaldehyde (5% Tween 80 in saline). BK and PGE2 were prepared in absolute ethanol and the solution used in the animals did not exceed 0.5% of ethanol in saline. All other drugs were made in physiological saline (0.9% NaCl solution).

### Statistical Analysis

All data are expressed as mean ± SEM of 4–6 animals/group and are representative of two independent experiments. A statistical comparison of the data was performed by two-way ANOVA followed by Bonferroni’s *post hoc* test or one-way ANOVA followed by Newman–Keuls’s test. *P-values* less than 0.05 (*P* < 0.05 or less) were considered significant. Statistical analyses were performed using GraphPad Prism 6 software (GraphPad Software Inc., San Diego, CA, United States).

## Results

### Single Treatment with Simvastatin Inhibits Mechanical Hyperalgesia in a Model of CRPS-I

**FIGURE 1:**
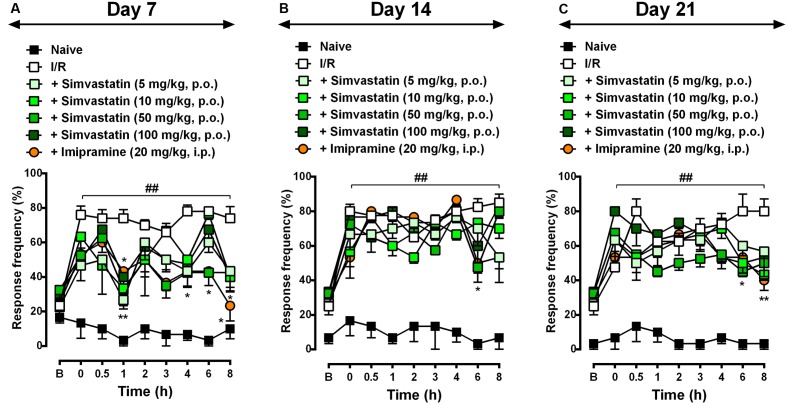
Simvastatin single treatment reduced mechanical hyperalgesia during CPIP. CPIP was induced via a 3-h hindpaw I/R injury, and animals treated with different doses of simvastatin (5, 10, 50, and 100 mg/kg, 1x/day, p.o.) evaluated at 7 **(A)**, 14 **(B)**, and 21 **(C)** days after nerve injury. Imipramine (20 mg/kg, i.p.) was used as positive control drug. Response of frequency of the ipsilateral withdrawal thresholds assessed at several time-points by von Frey hair test. **(B)** Baseline withdrawal threshold before I/R injury. Data are expressed as mean ± SEM (*n* = 6/group), and are representative of two independent experiments. ^##^*P* < 0.001 vs. naïve group, ^∗^*P* < 0.05 and ^∗∗^*P* < 0.001 vs. I/R group (two-way ANOVA followed by Bonferroni’s test).

Different reports have showed that statins presents its effects beyond dyslipidemia possibly through cholesterol-independent or pleiotropic effects (Palinski, 2001; Oesterle et al., 2017). Herein, we investigated simvastatin effect on CRPS-I, using a CPIP model, which showed inflammatory and neuropathic stages. In this set of experiments, we evaluated simvastatin single treatment effect on day 7 (**Figure 1A**), 14 (**Figure 1B**), and 21 (**Figure 1C**) after I/R procedure. We demonstrated that simvastatin single treatment inhibited mechanical hyperalgesia in the CRPS-I model (**Figure 1**). We found that in all evaluated days, I/R group showed long lasting and pronounced hyperalgesia when compared to naïve group at 0.5, 1, 2, 3, 4, 6, and 8 h (**Figure 1**). Interestingly, simvastatin treated-groups (single oral administration) inhibited mechanical hyperalgesia compared to I/R group: (i) at 1 h, simvastatin 5, 10, 50, and 100 mg/kg groups; (ii) at 4 and 6 h, simvastatin 50 mg/kg; and (iii) at 8 h, simvastatin 100 mg/kg and imipramine group (**Figure 1A**; ANOVA row [*F*(8,166) = 12.53, *P* < 0.0001^∗^], column [*F*(6,166) = 57.23, *P* < 0.0001^∗^], and interaction [*F*(48,166) = 1.96, *P* = 0.001^∗^] effects). On day 14, ANOVA showed row [*F*(8,144) = 28.48, *P* < 0.0001^∗^], column [*F*(6,144) = 102.40, *P* < 0.0001^∗^], and interaction [*F*(48,144) = 2.41, *P* < 0.0001^∗^] effects. The *post hoc* analysis demonstrated that at 6 h, only 50 mg/kg simvastatin group displayed inhibition of mechanical hyperalgesia when compared to I/R group (**Figure 1B**). On day 21, simvastatin treated-group showed inhibition of mechanical hyperalgesia compared to I/R group: (i) at 0.5 h, 100 mg/kg simvastatin group; (ii) at 6 h, 50 mg/kg simvastatin group, and (iii) at 8 h, 10 and 50 mg/kg simvastatin groups, as well as imipramine group (**Figure 1C**; ANOVA row [*F*(8,144) = 16.67, *P* < 0.0001^∗^], column [*F*(6,144) = 87.20, *P* < 0.0001^∗^], and interaction [*F*(48,144) = 2.36, *P* < 0.0001^∗^] effects). Based on these beneficial effects, a dose of 50 mg/kg of simvastatin was used in subsequent experiments to investigate some of the mechanisms underlying its antinociceptive effects.

### Repeated Treatment with Simvastatin Ameliorates Mechanical Hyperalgesia Induced by CRPS-I Model in Mice

**FIGURE 2:**
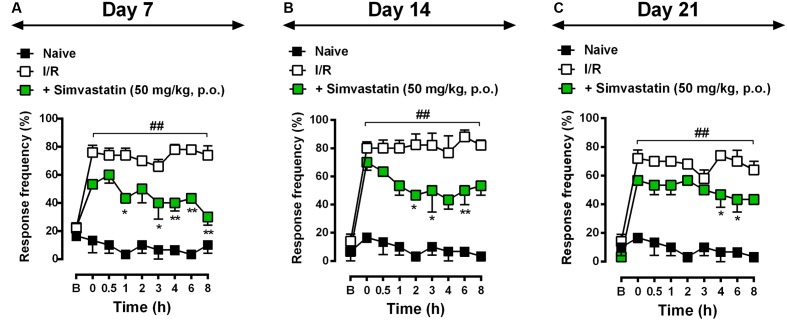
Simvastatin repeated treatment prevented the development of neuropathic pain induced by CPIP. CPIP was induced via a 3 h hindpaw I/R injury, and animals treated with simvastatin (50 mg/kg, 1x/day, p.o.) evaluated at 7 **(A)**, 14 **(B)**, and 21 **(C)** days after nerve injury. Imipramine (20 mg/kg, i.p.) was used as positive control drug. Response of frequency of the ipsilateral withdrawal thresholds assessed at several time-points by von Frey hair test. **(B)** Baseline withdrawal threshold before I/R injury. Data are expressed as mean ± SEM (*n* = 6/group), and are representative of two independent experiments. ^##^*P* < 0.001 vs. naïve group, ^∗^*P* < 0.05 and ^∗∗^*P* < 0.001 vs. I/R group (two-way ANOVA followed by Bonferroni’s test).

To evaluate the potential therapeutic effects of simvastatin on I/R-induced CPIP, mice were treated daily for 21 days with either simvastatin (50 mg/kg per day, p.o.) or vehicle, starting immediately after I/R procedure. Animals were evaluated on day 7 (**Figure 2A**), 14 (**Figure 2B**), and 21 (**Figure 2C**) post-I/R induction. In response to stimulation with von Frey filaments on the right hindpaw, untreated I/R mice showed long-lasting, pronounced and increased frequency response compared to naïve control group at 0.5, 1, 2, 3, 4, 6, and 8 h after induction (**Figure 2**). On day 7, simvastatin treatment prevented mechanical hyperalgesia compared to I/R group at 1, 3, 4, 6, and 8 h after nerve injury (**Figure 2A**; ANOVA row [*F*(8,73) = 6.84, *P* < 0.0001^∗^], column [*F*(2,73) = 272.90, *P* < 0.0001^∗^], and interaction [*F*(16,73) = 4.54, *P* < 0.0001^∗^] effects). On day 14, treatment with simvastatin (50 mg/kg, p.o.) notably attenuated mechanical hyperalgesia induced by I/R at 2, 3, 4, and 6 h after nerve injury (**Figure 2B**; ANOVA row [*F*(8,67) = 12.23, *P* < 0.0001^∗^], column [*F*(2,67) = 218.90, *P* < 0.0001^∗^], and interaction [*F*(16,67) = 4.45, *P* = 0.0002^∗^] effects). Moreover, on day 21, simvastatin oral treatment markedly reduced mechanical hyperalgesia compared to I/R group at 4 and 6 h after nerve injury (**Figure 2C**; ANOVA row [*F*(8,72) = 13.12, *P* < 0.0001^∗^], column [*F*(2,72) = 224.30, *P* < 0.0001^∗^], and interaction [*F*(16,72) = 3.75, *P* < 0.0001^∗^] effects). Taken together, our data indicates that simvastatin (single and, mainly, repeated treatment) was able to extinguish mechanical hyperalgesia induced by I/R model.

### Simvastatin Did Not Prevents Inflammatory Stages during CRPS-I Model

**FIGURE 3:**
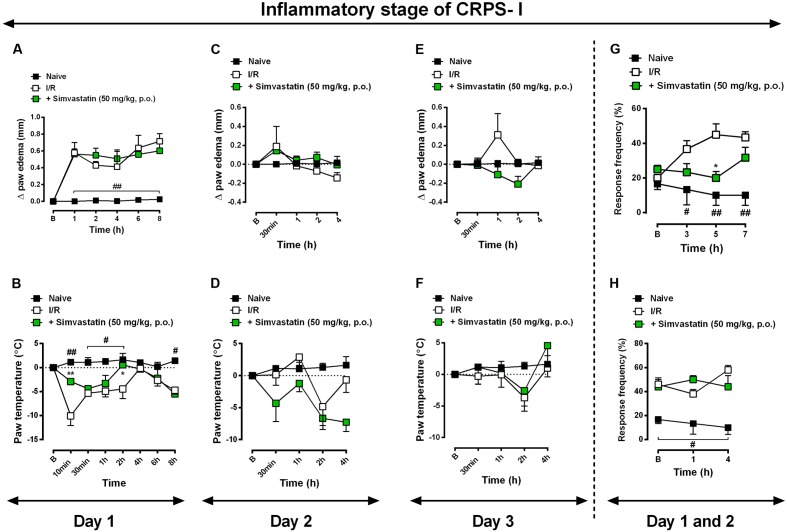
Simvastatin repeated treatment failed to reduce inflammatory signs induced by CRPS-I model. CPIP was induced via a 3 h hindpaw I/R injury, paw oedema **(A,C,E)** and paw temperature **(B,D,F)** on day 2 and 3, respectively, as well as mechanical hyperalgesia – day 1 **(G)** and day 2 **(H)** – were evaluated in the naïve group, untreated I/R group, and in mice treated with simvastatin (50 mg/kg, 1x/day, p.o.). Response of frequency of the ipsilateral withdrawal thresholds assessed at several time-points by von Frey hair test. **(B)** Baseline paws temperature oedema and withdrawal threshold before I/R injury. Data are expressed as mean ± SEM (*n* = 6/group), and are representative of two independent experiments. ^#^*P* < 0.05 and ^##^*P* < 0.001 vs. naïve group, ^∗^*P* < 0.05 vs. I/R group (two-way ANOVA followed by Bonferroni’s test).

Statins may exert anti-inflammatory action through inhibition of microglia and astrocytes cells, according to previously described (Cordle and Landreth, 2005; Lindberg et al., 2005; Li et al., 2009). More precisely, simvastatin have showed its anti-inflammatory properties in different other preclinical studies (Higuita-Castro et al., 2016; Wang et al., 2016; Barbosa et al., 2017). In addition, Qiu and colleagues (Qiu et al., 2016) showed that simvastatin attenuated neuropathic pain through inhibition of RhoA/LIMK/Cofilin pathway, which is activated after chronic constriction injury and related to actin dynamic regulation (Qiu et al., 2016). As expected, inflammatory symptoms, such as oedema (**Figures 3A,C,E**), paw temperature (**Figures 3B,D,F**) and hypersensitivity (**Figures 3G,H**) appeared on day 1, 2, and 3 post-I/R-induction, respectively. Interestingly, simvastatin treatment re-established the temperature dysfunction to control levels on day 1 at 10 and 120 min after I/R (**Figure 3B**; ANOVA row [*F*(7,93) = 4.54, *P* = 0.0002^∗^], column [*F*(2,93) = 30.39, *P* = 0.0001^∗^], and interaction [*F*(14,93) = 2.98, *P* = 0.0008^∗^] effects). Moreover, oral treatment with simvastatin (50 mg/kg, 1x/day) significantly reduced mechanical hyperalgesia induced by I/R induction on day 1 for up to 5 h (**Figure 3G**; ANOVA row [*F*(3,48) = 1.19, *P* = 0.3223], column [*F*(2,48) = 20.33, *P* < 0.0001^∗^], and interaction [*F*(6,48) = 2.90, *P* = 0.0172^∗^] effects), although failed to inhibit hyperalgesia on day 2. This collection of data demonstrates that simvastatin was able to abolish mechanical hiperalgesia, but it was unable to prevent inflammatory symptoms induced by I/R induction, when tested at the same schedule of treatment used during neurogenic neuroinflammation (**Figure 3**).

### Involvement of TRPM8 Channel on Antinociceptive Effect of Simvastatin

**FIGURE 4:**
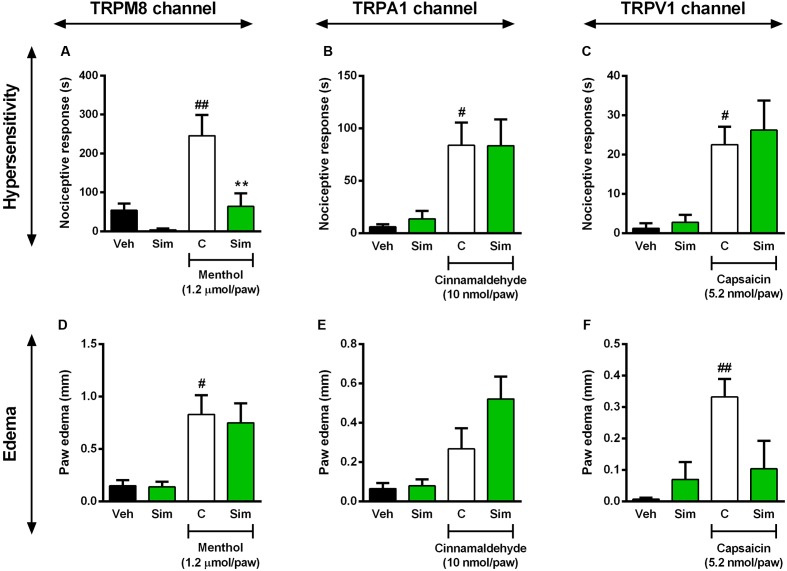
Effect of simvastatin on nociception and paw oedema response induced by intraplantar injection of menthol (TRPM8 activator, **A,D**), cinnamaldehyde (TRPA1 activator, **B,E**) and capsaicin (TRPV1 activator, **C,F**) in mice, respectively. Animals were pre-treated with simvastatin (50 mg/kg, p.o.) or vehicle (saline solution, 10 ml/kg, p.o., control group) 1 h before the algogenic injections. Data are expressed as mean ± SEM (*n* = 5/group), and are representative of two independent experiments. ^#^*P* < 0.05 and ^##^*P* < 0.001 vs. vehicle group, ^∗^*P* < 0.05 vs. untreated-control group (one-way ANOVA followed by Newman–Keuls).

There are markedly evidences showing that transient receptor potential (TRP) displays an important role in the detection of noxious stimuli (Numazaki and Tominaga, 2004; Tominaga, 2007). More precisely, TRPM8 is a TRP channel member that senses cold stimuli (McKemy et al., 2002; Peier et al., 2002) and seems to be closed involved with modulation of nociception (Bautista et al., 2007). Next, we sought to verify whether simvastatin could selectively modulate TRPs channels, especially TRPM8, TRPA1 and TRPV1, and for this purpose we used different approaches. First we evaluated simvastatin effects on mechanical hypersensitivity induced by menthol, a selective TRPM8 activator. Intraplantar injection of menthol (1.2 nmol/paw) induced a pronounced mechanical hypersensitivity and oedema, when compared to baseline values (**Figures 4A,D**). Importantly, pre-treatment with simvastatin (50 mg/kg, p.o.; 1 h prior) significantly reduced menthol-induced hyperalgesic response (inhibition of 73.87%. **Figure 4A**; ANOVA showed a significant treatment effect [*F*(3,13) = 9.68, *P* = 0.0013^∗^]), although failed to inhibit menthol-induced oedema (**Figure 4D**). Aiming to rule out the possibility of other TRPs involvement in the antinociceptive effects of simvastatin, we accessed in another set of experiments, the simvastatin effects in the cinnamaldehyde – (a TRPA1 channel activator) and the capsaicin-induced hypersensitivity and oedema (a TRPV1 agonist). The intraplantar injection of cinnamaldehyde or capsaicin induced a marked and long-lasting enhancement of response frequency to von Frey hair applications compared to naïve mice (**Figures 4B,C**; ANOVA [*F*(3,13) = 4.39, *P* = 0.0242^∗^], and [*F*(3,13) = 6.52, *P* = 0.0062^∗^]), as well as oedema formation (**Figures 4E,F**; ANOVA [*F*(3,14) = 5.738, *P* = 0.0089^∗^], and [*F*(3,14) = 5.044, *P* = 0.0141^∗^]), respectively. Interestingly, oral treatment with simvastatin (50 mg/kg; 1 h prior) did not alter the response of frequency of the ipsilateral withdrawal thresholds (**Figures 4B,C**) and oedema formation (**Figures 4E,F**) induced by a TRPA1 or TRPV1 signaling pathway activator, respectively. Altogether, this set of data suggests that simvastatin could selectively modulate TRPM8 channel activation producing a TRPA1/TRPV1-independent antinociception.

### Role of ASIC Channel and BK Signaling Pathway in the Anti-hyperalgesic Effect Caused by Simvastatin

**FIGURE 5:**
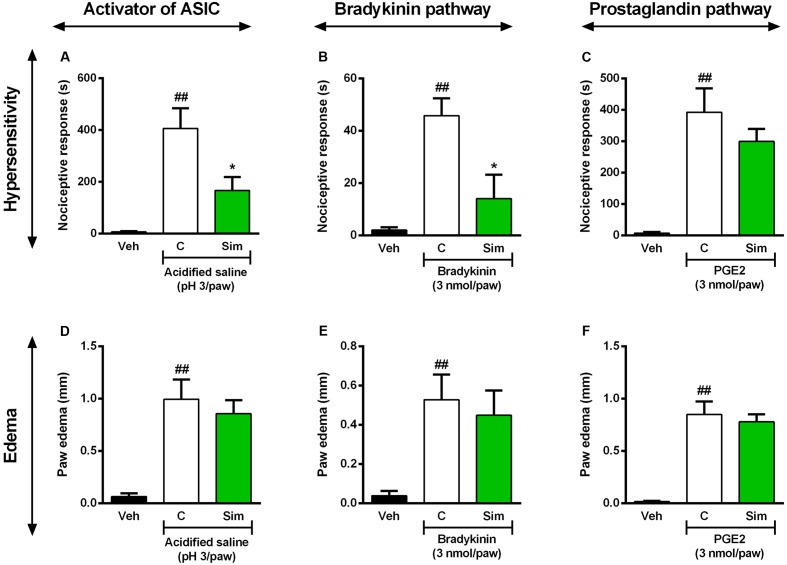
Effect of simvastatin on licking and paw oedema response induced by intraplantar injection of acidified saline (ASIC activator, **A,D**), BK **(B,E)** and PGE2 **(C,F)** in mice, respectively. Animals were pre-treated with simvastatin (50 mg/kg, p.o.) or vehicle (saline solution, 10 ml/kg, p.o., control group) 1 h before the algogenic injections. Data are expressed as mean ± SEM (*n* = 5/group), and are representative of two independent experiments. ^##^*P* < 0.001 vs. vehicle group, ^∗^*P* < 0.05 vs. untreated-control group (one-way ANOVA followed by Newman–Keuls).

Acid-sensing ion channels are widely expressed in peripheral and central nervous system and commonly activated by inflammatory pain mediators (Deval et al., 2010). It was previously demonstrated that upregulation of ASIC in spinal dorsal horn neurons contributed to inflammatory pain hypersensitivity (Duan et al., 2007). Furthermore, recently it was shown ASICs participation in the antinociceptive effect of curcumin during formalin-induced orofacial inflammatory model (Wu et al., 2017). In this set of experiments, we investigated whether the same oral treatment with simvastatin could inhibit the hypersensitivity and oedema induced by ASIC activator in mice. The results of **Figure 5** demonstrate that acidified saline (pH 3/paw) induces a significant decrease in mechanical nociceptive thresholds (**Figure 5A**; ANOVA [*F*(2,9) = 13.81, *P* = 0.0018^∗^]) and oedema (**Figure 5D**; ANOVA [*F*(2,11) = 11.34, *P* = 0.0021^∗^]) observed in the paw ipsilateral when compared with naïve mice. Interestingly, oral treatment with simvastatin (50 mg/kg) 1 h before significantly reduced mechanical hyperalgesia induced by acidified saline (pH 3/paw) in mice (inhibition of 59.11%, **Figure 5A**), and did not inhibit oedema formation (**Figure 5D**). Moreover, after tissue damage, nociceptive afferents and/or non-neural cells release and accumulate endogenous algesic mediators, such as BK and PGE2 (Julius and Basbaum, 2001; Basbaum et al., 2009). Into inflammatory conditions, BK signaling is also potentiated by protons that activate ASICs (Voilley et al., 2001; Mamet et al., 2002). This cascade contributes then to the maintenance of the painful process and consequently promotes hyperalgesia or allodynia (Calixto et al., 2001; Ikeda et al., 2001; Julius and Basbaum, 2001). To extend our above findings, we next assessed whether oral treatment with simvastatin could inhibit mechanical hypersensitivity and oedema formation evoked by BK or PGE2 in mice. As demonstrated in **Figure 5**, intraplantar injection of BK (3 nmol/paw) or PGE2 (3 nmol/paw) induced a significant decrease of mechanical withdrawal threshold in mice (**Figures 5B,C**; ANOVA [*F*(2,10) = 12.28, *P* = 0.0020^∗^] and [*F*(2,11) = 13.10, *P* = 0.0012^∗^]), as well as increase paw oedema after injection (**Figures 5E,F**; ANOVA [*F*(2,11) = 5.00, *P* = 0.0285^∗^] and [*F*(2,11) = 24.28, *P* < 0.0001^∗^]), respectively. In comparison to untreated group, simvastatin (50 mg/kg, p.o.) markedly abolished mechanical hypersensitivity induced by BK (inhibition of 69.43%, **Figure 5B**) through independent-oedema mechanism (**Figure 5E**). Furthermore, simvastatin did not recover sensory pathways (**Figure 5C**) and inflammatory signs (**Figure 5F**) induced by PGE2 (*P* > 0.05).

### Evaluation of Noradrenergic, Serotoninergic and Dopaminergic Systems in Antinociceptive Effect Caused by Simvastatin

**FIGURE 6:**
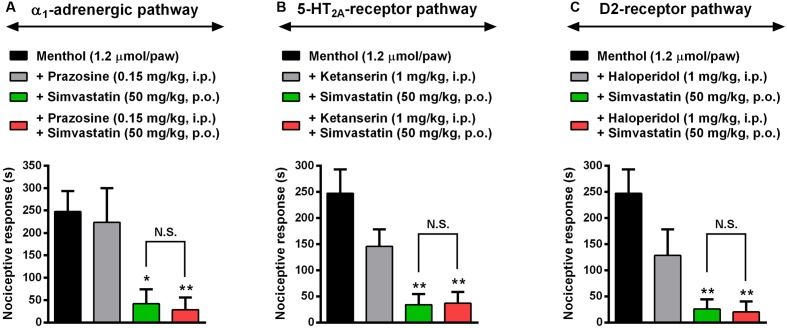
Evaluation of noradrenergic, serotoninergic and dopaminergic systems in antihypersensitivity effect caused by simvastatin. Intraperitoneal (i.p.) pre-treatment with prazosin (0.15 mg/kg, a selective α1-adrenergic receptor antagonist, **A**), ketanserin (1 mg/kg, a selective 5-HT2A receptor antagonist, **B**) or haloperidol (1 mg/kg, a selective D2 receptor antagonist, **C**) on the antihypersensitivity effect of simvastatin (50 mg/kg, p.o.) during menthol-induced paw nociception. Data are expressed as mean ± SEM (n = 4/group), and are representative of two independent experiments. ^∗^*P* < 0.05 and ^∗∗^*P* < 0.001 vs. menthol-control group (one-way ANOVA followed by Newman–Keuls). N.S., not significant.

Considering the remarkable antinociceptive effect caused by simvastatin in the menthol-induced inflammatory model of pain, we next evaluated some of the descending pain circuits, especially noradrenergic, serotoninergic and dopaminergic systems, involved in simvastatin antinociception effects. As described previously, menthol-induced a pronounced mechanical hypersensitivity (*P* < 0.001), and simvastatin treatment completely blocked nociceptive response induced by TRPM8-activator (**Figure 6**). Clinically relevant, prazosin (a selective α1-adrenergic receptor antagonist, 0.15 mg/kg, i.p.) or ketanserin (a selective 5-HT2A receptor antagonist, 1 mg/kg) or haloperidol (a selective D2 receptor antagonist, 1 mg/kg) did not significantly change the antinociception caused by simvastatin in menthol-induced mechanical hyperalgesia (**Figure 6**). Furthermore, prazosin (**Figure 6A**), ketanserin (**Figure 6B**) or haloperidol (**Figure 6C**) without simvastatin had no effect on the increased response frequency similarly to menthol-untreated animals. These data, allied to those presented before, allow us to suggest that antinociceptive effect displayed by simvastatin upon neuropathic and inflammatory hyperalgesia might be related, at least in part, on its ability to modulate TRPM8 channel and ASICs/BK signaling pathways, without affecting descending pain circuits. However, further experiments are needed to clarify whether simvastatin treatment could have some effect on other descending pain control system, such as endogenous opioids pathways.

## Discussion

This study was outlined to investigate simvastatin effects on CRPS-I model. Here, we have used the CPIP model to study simvastatin effects in CRPS-I, which symptoms mimicking after the prolonged hindpaw I/R (for more detail see Coderre et al., 2004). According to our results, single treatment with simvastatin inhibited mechanical hyperalgesia after 7, 14, and 21 days post-CPIP model. Moreover, simvastatin ameliorates mechanical hyperalgesia on day 7, 14, and 21 after I/R induction, when administrated in the repeated treatment. It demonstrates that simvastatin effect in reducing mechanical hyperalgesia had a particular profile in each time-point of the CRPS-I model. Dose-related effects of simvastatin were already seen in other neuropathic pain models (Bhalla et al., 2015). These results are in agreement with studies in rodent models demonstrating that simvastatin ameliorated neuropathic pain (Ohsawa et al., 2016) and blocked formalin-induced nociceptive behaviors in mice (Chen et al., 2013).

In addition, our findings showed that simvastatin did not prevent inflammatory phase during CRPS-I model. It was surprisingly, since statins may exert anti-inflammatory action through inhibition of glial cells (Cordle and Landreth, 2005; Lindberg et al., 2005; Li et al., 2009) showing its role as potential therapeutic agents in neuroinflammatory disorders (Stüve et al., 2003). More precisely, simvastatin have showed its anti-inflammatory properties in several other preclinical studies (Higuita-Castro et al., 2016; Wang et al., 2016; Barbosa et al., 2017). Qiu et al. (2016) showed that simvastatin attenuated neuropathic pain by inhibiting the RhoA/LIMK/Cofilin pathway, which is activated after chronic constriction injury and related to actin dynamic regulation. Also, simvastatin attenuated formalin-induced nociceptive behaviors through inhibition of microglial RhoA and p38 MAPK activation (Chen et al., 2013). In fact, neurogenic neuroinflammation is a key pathophysiological mechanism in CRPS (Littlejohn, 2015), which is caused by release of pro-inflammatory peptides – including substance P, calcitonin gene relates peptide (CGRP) and neurokinin A – from the peripheral nerve involved in nociception (Holzer, 1998). Exacerbation of these neuroinflammatory mechanisms is important in the early stages of CRPS, and can persist over time to contribute to ongoing clinical symptoms. Moreover, these neuropeptides directly attract and activate cell types involved in both innate (neutrophils, mast cells, dendritic cells, and keratinocytes) and adaptive (T lymphocytes) immunity (Chiu et al., 2012). These neuropeptides sensitize other nearby nociceptive terminals, such as Aδ myelinated fibers, resulting in further amplification of inflammatory changes in the affected site (Birklein and Schmelz, 2008). Based on these results, it is tempting to speculate that disease attenuation induced by simvastatin observed in our experiment might be related to the down regulation of release and/or expression of neuropeptides through an immune response-independent pathway. Thus, whether statins – especially simvastatin – directly affect neurogenic neuroinflammation and consequently inflammatory phase during CRPS-I model is an important question that remains to be further addressed.

Regarding underlying mechanisms of analgesia, we next evaluated some of the intracellular pathways involved in simvastatin antinociception effects, especially ascending pain transmission system. There are markedly evidences in the literature that TRP and ASIC display important role in detection of noxious stimuli (Caterina and Julius, 1999; Numazaki and Tominaga, 2004; Tominaga, 2007; Simões et al., 2017). Particularly, TRP channels are a large family of non-selective cation channels, which includes TRPV1, TRPV3, TRPA1, TRPM8 and others expressed in different populations of primary afferent neurons (Huang et al., 2012). TRPM8 channels are involved in the modulation of nociception and are expressed in about 15 % of all somatosensory neurons, predominantly unmyelinated C-fibers, and in a mechanoreceptors (AM-fibers) subset of myelinated Aδ fibers (Bautista et al., 2007). These have been extended by McKemy et al. (2002) and Peier et al. (2002), who demonstrated that TRPM8 is a TRP channel member that senses cold stimuli and menthol (McKemy et al., 2002; Peier et al., 2002). In this sense, we have found that TRPM8 channel is involved in the antinociceptive effect of simvastatin. Takashima et al. (2007) and Dhaka et al. (2008) reported that 38 and 18 % of C-fibers co-express TRPM8 and TRPV1, respectively, suggesting that both channels can be co-expressed in the same C-fiber. In addition, TRPV1 also showed different functions as a polymodal noxious receptor at peripheral nerve terminals (Premkumar and Abooj, 2013). After TRPV1 activation, many other signaling pathways can be stimulated due to TRPV1 multiple phosphorylation sites in its amino acid sequence for protein kinase C (PKC) (Bhave et al., 2003; Premkumar et al., 2004), protein kinase A (PKA) (Bhave et al., 2002) and Ca^2+^/calmodulin-dependent protein kinase II (CaMKII). Altogether, in sensory neurons, these protein kinases can sensitize their receptors or amplify their responses by phosphorylating key residues in their structures or other proteins implicated in nociceptive signaling pathways (Wood et al., 1988; Premkumar and Ahern, 2000; Bhave et al., 2003). Here, simvastatin did not prevent hypersensitivity induced by capsaicin or cinnamaldehyde suggesting that its antinociceptive effect seem did not require TRPV1 or TRPA1 activation, respectively.

As TRPV1, the acid sensitive ion channels (ASICs) are other cationic channels activated under inflammatory pain conditions (Deval et al., 2010). Recent evidences suggest that ASIC and TRPV1 have complementary roles in the proton sensitivity of sensory neurons (Baggio et al., 2012). The pathways for pain signaling comprise peripheral polymodal nociceptors sensitive to protons, which depolarize sensory neurons by directly activating cationic channels (Coutaux et al., 2005). Moreover, protons that activate ASICs potentiate different pain mediators present in the inflammatory site [principally nerve growth factor (NGF), serotonin, interleukin-1β, and BK] (Voilley et al., 2001; Mamet et al., 2002). This complex cascade contributes then to the maintenance of the painful process and to promote hyperalgesia and allodynia (Calixto et al., 2001; Ikeda et al., 2001; Julius and Basbaum, 2001). Thus, to further investigate the mechanisms underlying the simvastatin actions, we next investigate whether or not its effects were also linked to ASIC channels and BK signaling pathways. Here, using an acute-lasting hyperalgesic response model, we found that simvastatin caused significant inhibition of the inflammatory pain induced by acidified saline and BK, although failed to inhibit PGE2-induced mechanical hyperalgesia. This set of data suggests simvastatin could selectively modulate TRPM8/ASIC channels and BK pathways activation producing a TRPV1/TRPA1/PGE2-independent antinociception.

Considering the involvement of TRPM8 channel on simvastatin antinociceptive effect, next we sought to verify whether, besides selectively modulate evoked activity of C and Aδ fibers, simvastatin could initiate the descending pain controls from the brainstem during menthol-induced mechanical hyperalgesia. There are some evidences exploring the relation between simvastatin and its central effects, such as: (i) altering dopamine D1 and D2 receptors, Wang et al. (2005, 2006); (ii) decreasing serotonin transporter (SERT) activity and membrane microviscosity (Jiang et al., 2015; Vevera et al., 2016), or in other animal models; (iii) anxiety and depression (Santos et al., 2012); (iv) in a rat model of pulmonary artery hypertension (Jiang et al., 2015); however, a relationship between simvastatin and noradrenergic, serotoninergic or dopaminergic systems during pain conditions is currently poorly understood. Moreover, Pleiner et al. (2004) demonstrated that high-dose of simvastatin showed significant vasoprotective properties during endotoxemia, as well as it was able to prevent vascular hyporeactivity during inflammatory response. Here, our results showed that simvastatin action inhibiting menthol-induced mechanical hyperalgesia was independent of both α1-adrenergic, 5-HT2A receptor, and D2 receptor pathways, since neither prazosin nor ketanserin nor haloperidol, respectively, were capable to interfere with antinociceptive effects of simvastatin. Altogether, this set of data reinforces the idea of ascending pain signaling (TRPM8/ASIC/BK pathways expressed in the primary sensory neuron) is, at least in part, involved in the mechanical antinociception elicited by simvastatin.

## Conclusion

**FIGURE 7:**
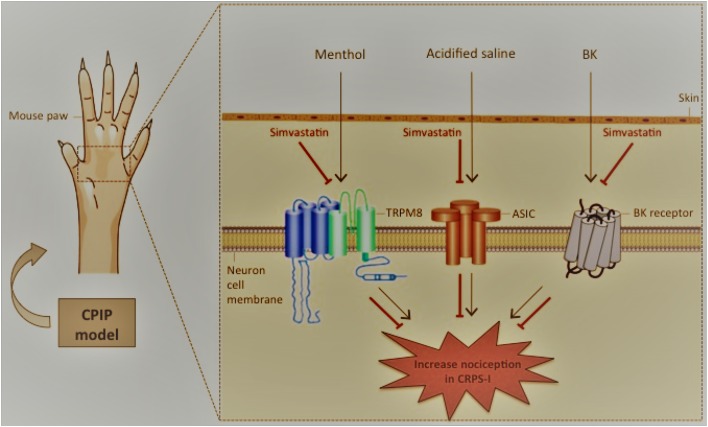
Schematic diagrams illustrating the beneficial effects of simvastatin during experimental model of CRPS-I in mice. Simvastatin – inhibitor of 3-hydroxy-methyl-glutaryl coenzyme A (HMG-CoA) reductase – emerges as a possible new venue for an effective CRPS-I treatment, in monotherapy or in combination with existing therapies. Here, simvastatin: (i) inhibited mechanical hyperalgesia induced by CPIP model in single and repeated treatment schedules; (ii) did not prevent inflammatory signs during CPIP model; (iii) required the TRPM8 and ASIC channel, as well BK signaling pathways in its anti-hyperalgesic effect. CPIP, chronic post-ischemia pain; BK, bradykinin; CRPS-I, complex regional pain syndrome type I. Inhibition: ⊣.

In summary, we demonstrated that simvastatin exert a pleiotropic action modulating mechanical hyperalgesia and providing anti-hyperalgesic effect during CPIP model. In addition, we demonstrated that simvastatin antinociceptive effects are potentially associated with TRPM8 and ASIC channels, as well as BK signaling pathways. Moreover, we demonstrated that the mechanisms involved in simvastatin-mediated antinociceptive action appear to be independent of descending pain control system, especially, noradrenergic, serotonin and dopaminergic (see proposed scheme in **Figure 7**). Therefore, simvastatin open-up new standpoint in the development of innovative analgesic drugs for the treatment of persistent pain, including CRPS-I.

## Declaration of Transparency and Scientific Rigor

This declaration acknowledges that this paper adheres to the principles for transparent reporting and scientific rigor of pre-clinical research recommended by funding agencies, publishers and other organizations engaged with supporting research.

## Author Contributions

GV and RD conceived and designed this research; GV, EG, TG, LL, and RD designed, performed the experiments and analyzed the data; GV, JC, MC, and RD interpreted the results of experiments; JC and RD prepared the figures and drafted the manuscript; JC, MC, and RD edited and revised the manuscript; RD approved the final version of the manuscript.

## Conflict of Interest Statement

The authors declare that the research was conducted in the absence of any commercial or financial relationships that could be construed as a potential conflict of interest.

## Abbreviations

ANOVA: analysis of variance
ASICs: acid-sensing ion channels
BK: bradykinin
CPIP: chronic post-ischemia pain
CRPS-I: complex regional pain syndrome-type I
HMG-CoA: 3-hydroxy-methylglutaryl coenzyme A
IR: ischemia and reperfusion
PGE2: prostaglandin E2
TRPA1: transient receptor potential ankyrin 1
TRPM8: transient receptor potential cation channel subfamily M member 8
TRPs: transient receptor potential channels
TRPV1: transient receptor potential vanilloid 1
TRPV3: transient receptor potential cation channel subfamily V member 3
MAPK: mitogen activated protein kinases
